# Neutrophils From Patients With Invasive Candidiasis Are Inhibited by *Candida albicans* Biofilms

**DOI:** 10.3389/fimmu.2020.587956

**Published:** 2020-12-03

**Authors:** John F. Kernien, Chad J. Johnson, Meg L. Bayless, Jack F. Chovanec, Jeniel E. Nett

**Affiliations:** ^1^Department of Medicine, University of Wisconsin-Madison, Madison, WI, United States; ^2^Department of Medical Microbiology and Immunology, University of Wisconsin-Madison, Madison, WI, United States

**Keywords:** *Candida*, biofilm, neutrophil, neutrophil extracellular trap, patients, invasive candidiasis, reactive oxygen species

## Abstract

Invasive candidiasis frequently involves medical device placement. On the surfaces of these devices, *Candida* can form biofilms and proliferate in adherent layers of fungal cells surrounded by a protective extracellular matrix. Due in part to this extracellular matrix, biofilms resist host defenses and antifungal drugs. Previous work (using neutrophils from healthy donors) found that one mechanism employed to resist host defenses involves the inhibition of neutrophil extracellular traps (NET) formation. NETs contain nuclear DNA, as well as antimicrobial proteins that can ensnare pathogens too large or aggregated to be effectively killed by phagocytosis. Given that these neutrophil structures are anticipated to have activity against the large aggregates of *C. albicans* biofilms, understanding the role of this inhibition in patients could provide insight into new treatment strategies. However, prior work has not included patients. Here, we examine NET formation by neutrophils collected from patients with invasive candidiasis. When compared to neutrophils from healthy participants, we show that patient neutrophils exhibit a heightened background level of NET release and respond to a positive stimulus by producing 100% more NETs. However, despite these physiologic differences, patient neutrophil responses to *C. albicans* were similar to healthy neutrophils. For both groups, planktonic cells induce strong NET release and biofilms inhibit NET formation. These results show that a mechanism of immune evasion for fungal biofilms translates to the clinical setting.

## Introduction

*Candida albicans*, a widespread nosocomial fungal pathogen, is an avid biofilm-former and a frequent cause of invasive fungal infection (1). On the surface of medical devices, such as vascular catheters, *Candida* spp. adopt a biofilm lifestyle and grow as adherent communities encased in an extracellular matrix with protective properties (2). *Candida* biofilms infections are notoriously difficult to treat, as they resist high levels of antifungal drugs and withstand host defenses (3–10). Despite advancements in antifungal therapies and diagnostics, the mortality for *Candida*-associated bloodstream infection remains exceedingly high, near 30% (1, 11).

Neutrophils are essential for eradicating many fungal infections, including invasive candidiasis (12–15). Neutropenic patients are at high risk for critical disease, and those remaining neutropenic are more likely to relapse (16). However, in the clinical setting, neutrophils are ineffective at controlling *Candida* device-associated biofilm infections. Eradication of infection most often requires device removal, even for patients with normal numbers of neutrophils (11). Moreover, mortality rates increase for patients when biofilm-infected devices are retained (1, 11).

*Ex vivo*, *C. albicans* biofilms are approximately 5-fold more resistant to killing by neutrophils when compared to non-biofilm, planktonic cells (7, 8, 17, 18). When no biofilm is present, neutrophils phagocytose the smaller yeast form of *C. albicans* and release neutrophil extracellular traps (NETs) in response to the larger, elongated hyphal form (19, 20). NETs consist of extracellular web-like structures of DNA associated with citrullinated histones and other antimicrobial proteins that contain and kill pathogens. These structures exhibit antifungal activity against both yeast and hyphae (19, 21, 22). NETs are anticipated to be crucial for controlling *Candida* infections because they can target hyphae, which are too large to be phagocytosed (20). Given their role in combatting hyphae and their activity against aggregative pathogens, the formation of NETs would seem to be an ideal response against *Candida* biofilm. However, neutrophils (collected from healthy study participants) fail to release NETs in response to *Candida* biofilms (18, 23, 24). This impaired innate immune response likely contributes to the resilient nature of device-associated *Candida* infections.

While our understanding neutrophil-*Candida* biofilm interactions has advanced significantly using healthy participant neutrophils, studies have not delineated the relevance of these interactions for patients with invasive candidiasis. We considered that infection with *Candida* may influence neutrophil responses. The conventional concept poses that neutrophils are uniformly short-lived, terminally differentiated cells. However, recent investigations reveal phenomena of significant neutrophil plasticity and heterogeneity in a variety of clinical settings (25–27). Here we compare neutrophil responses between healthy participants and those with invasive candidiasis, focusing on NET formation.

## Methods

### Organisms and Inoculum

*C. albicans* (SC5314) was stored in 15% glycerol stock (vol/vol) at -80°C and plated on yeast extract-peptone-dextrose agar (YPD-agar) plates (1% yeast extract, 2% peptone, 2% dextrose, 2% agar) prior to experiments. Cultures were grown overnight in YPD media (1% yeast extract, 2% peptone, and 2% dextrose) at 200 RPM at 30°C in an orbital shaker. For experiments using biofilms, *C. albicans* was resuspended in RPMI-MOPS at a concentration of 1.5 × 10^6^ cells/ml and added at volumes specified in individual assays, followed by a 24 h incubation at 37°C. For experiments with planktonic cells, 1 ml of an overnight culture was added to 10 ml fresh YPD broth for 2 h at 180 RPM at 30°C. Following the incubation, the planktonic *C. albicans* cells were washed twice with phosphate-buffered saline (-calcium and -magnesium) DPBS (Hyclone Laboratories Inc., Logan, UT) and counted with a hemocytometer. For Sytox Green and reactive oxygen species (ROS) production assays, a burden of planktonic cells (3 × 10^6^ cells/well) equivalent to biofilm burden was established, as described previously (18). For other experiments, planktonic cells were added at concentrations described below.

### Study Participants

We obtained blood samples from patients with invasive candidiasis, through protocol 2017-0685 approved by the University of Wisconsin Internal Review Board (IRB). Patients were eligible upon identification of *Candida* growing as a culture from a normally sterile site, in accordance with clinical diagnosis of invasive candidiasis (16). All patients were 18–89 and admitted for hospitalization at the University of Wisconsin Hospital. We collected blood samples following written informed consent. Patient characteristics are presented in **Table 1**. Blood from healthy participants was collected through protocol 2013-1758 approved by the University of Wisconsin IRB. Healthy participants were 18 years of age or older and the group included 9 males and 10 females.

**Table 1:** Characteristics of patients with invasive candidiasis.

Study ID	Age (yr)M/F	Admitting diagnosis	Major comorbidities	Patient location	Antifungal therapy at enrollment	Immune-suppressing therapy prior to enrollment	Central line	*Candida* sp(p). culture source
A	44 M	Catheter-associated bloodstream infection	Renal transplant recipient, short gut syndrome requiring total parenteral nutrition, hypertension	Medical ward	MFG	Tacrolimus Prednisone Mycophenolate mofetil	Yes	*C. glabrata*: Blood Catheter tip
B	61 M	Cholangitis with sepsis	Malignant neoplasm of head and neck, emphysema, recurrent bacteremia, choledocholithiasis	Medical ward	MFG	None	Yes	*C. krusei*: Bile, Biliary drain tip *C. albicans*: Biliary drain tip
C	80 F	Perforated sigmoid colon with abscess formation	End-stage renal disease, chronic pancreatitis, hypertension, left hemicolectomy with colostomy	Surgical intermediate care	MFG	None	No	*C. glabrata*: Abdominal fluid
D	68 M	Cholangitis, empyema	Renal transplant recipient, liver transplant recipient, diabetes mellitus	Surgical ward	FLC	Cyclosporine Mycophenolate sodium	No	*C. albicans*: Bile Pleural fluid
E	55 F	Small bowel obstruction, spinal osteomyelitis	Non-Hodgkin’s lymphoma in remission, congestive heart failure, diabetes mellitus	Surgical ward	None	None	Yes	*C. glabrata*: Abdominal fluid
F	51 M	Intra-abdominal abscess	Colon cancer, hydronephrosis, severe thrombocytopenia, diverticulitis with perforation	Surgical ward	FLC	None	Yes	*C. dubliniensis*: Abdominal abscess
G	72 M	Pyelonephritis	Acute kidney injury, hypertension, history of renal cell carcinoma	Surgical ward	FLC	None	No	*C. glabrata*: Renal pelvis Urine
H	67 F	Obstructing pyelonephritis, sepsis, demand myocardial ischemia	Hypertension, hypothyroidism, acute kidney injury, acute pulmonary edema, acute heart failure, rheumatoid arthritis	Medical ward	FLC MFG	Adalimumab	No	*C. albicans*: Blood Urine *C. glabrata*: Blood
I	22 F	Port site infection	Cystic fibrosis, diabetes mellitus, allergic bronchopulmonary aspergillosis	Critical care ward	MFG	None	Yes	*C. dubliniensis:* Port site
J	78 F	Liver abscess	Cholangiocarcinoma, hypertension, dyslipidemia	Medical ward	MFG	None	No	*C. albicans*: Liver abscess

### Neutrophil Isolation

We isolated primary human neutrophils from whole blood by negative antibody selection using the MACSxpress Neutrophil Isolation and MACSxpress Erythrocyte Depletion kit (Miltenyi Biotec Inc., Auburn, CA). Experiments with isolated neutrophils were performed in RPMI 1640 media (without phenol red) supplemented with 2% heat-inactivated fetal bovine serum (FBS) and glutamine (0.3 mg/ml). All incubations were at 37°C with 5% CO_2_. We collected neutrophils from up to 12 ml of blood, which resulted in 1.0 × 10^7^ to 6.1 × 10^7^ neutrophils per patient.

### Sytox Green Assays

For a quantitative measure of NET formation, we used Sytox Green assays, as described previously (18, 23). To measure NET release in the presence of biofilm, we grew *C. albicans* as a biofilm for 24 h in 96-well opaque plates. To measure NET formation in the presence of planktonic cells, we added 3 × 10^6^ planktonic cells/well prior to the addition of neutrophils. For a subset of experiments, phorbol myristate acetate (PMA) was added at a final concentration of 100 nM. Neutrophils were added at a final concentration of 2 × 10^5^ neutrophils/well. After a 4 h incubation, the cell-impermeable free DNA fluorescent dye Sytox Green (Life Technologies, Eugene, OR) was added and read at excitation 500 nm/emission 528 nm, as previously described (18, 28). In experiments with *C. albicans*, fluorescence for *C. albicans* biofilms or planktonic cells alone was analyzed and subtracted as background fluorescence.

### ROS Production Assays

To measure ROS production, we used an oxidative stress assay modified for biofilm (18, 23). Planktonic cells or biofilm were added to 96-well opaque plates, as described above. Neutrophils were stained at room temperature with fluorescent dye CM-H2DCFDA (Life Technologies, Eugene, OR) for 10 min in DPBS prior to addition to a 96-well opaque plate at a final concentration of 2 × 10^5^ neutrophils/well. For some experiments, PMA was added at a final concentration of 100 nM. Fluorescence was recorded prior to incubation at 37°C and at every 30 min for 4 h (excitation, 495 nm; emission, 527 nm). In experiments with *C. albicans*, fluorescence for *C. albicans* biofilms or planktonic cells alone was recorded and subtracted as background fluorescence.

### Scanning Electron Microscopy

For electron microscopy experiments, we utilized poly-L-lysine-treated plastic coverslips (13 mm, Thermonax plastic for cell culture). Neutrophil (5 × 10^5^) were added to coverslips containing *C. albicans* biofilm, planktonic cells, or PMA. For biofilm experiments, biofilms were grown on coverslips, as previously described (18, 23). Briefly, 1.5 × 10^6^ cells/ml *C. albicans* were applied to coverslips at 30°C for 30 min. After removing non-adherent cells, fresh RPMI-MOPS was added and biofilms were grown for 24 h at 37°C and then washed with DPBS. For planktonic interactions, planktonic cells were added to coverslips (1.5 × 10^7^ cells/coverslip) for 1 h prior to the addition of neutrophils. For a subset of experiments, PMA was included at a final concentration of 100 nM. Following a 4 h incubation, coverslips were processed for scanning electron microscopy, as described previously (18, 23). Briefly, samples were washed in DPBS and fixed overnight in a 4% formaldehyde and 1% glutaraldehyde solution in DPBS. Following fixation, samples were treated with 1% osmium tetroxide for 1 h, and then washed with DPBS. The samples then underwent ethanol dehydration, which was immediately followed with critical point drying. Samples were then placed on aluminum stubs and sputter-coated with 10 nm platinum. The samples were imaged at 3 kV by a LEO 1530 scanning electron microscope.

### Immunofluorescence Microscopy

We utilized immunofluorescence microscopy to visualize the location of neutrophil elastase. Neutrophils were analyzed in 8-well Ibidi devices. Briefly, neutrophils (1 × 10^5^/well) were added to wells containing biofilms, planktonic cells, or PMA (100 nM). To examine neutrophil-biofilm interactions, we seeded 100 µl of *C. albicans* in RPMI-MOPS at 1.5 × 10^6^ cells/ml and incubated for 24 h on a 45° incline to propagate biofilm. After 6 h, 50 µl of RPMI-MOPS was gently added to prevent biofilm growth at the air interface. Following 24 h of growth, biofilms were removed from incline and gently rinsed with DPBS prior adding neutrophils. For planktonic studies, we added 4 × 10^5^ planktonic *C. albicans* cells per device. Following a 4 h incubation, samples were fixed for 2 h in 1% formaldehyde in DPBS. Samples were then washed with DPBS and incubated with blocking buffer (2% w/v bovine serum albumin (BSA) and 0.02% v/v Tween 20 in DPBS) overnight at 4°C. Samples were exposed to anti-Neutrophil elastase rabbit pAb (MilliporeSigma, Burlington, MA) or anti-Histone 4 (citrulline 3) rabbit pAB (EMD Millipore, Temecula, CA) at a 1:200 dilution in binding buffer (0.1% BSA w/v and 0.005% v/v Tween 20 in DPBS) overnight at 4°C. Samples were washed 3× for 5 min in fresh binding buffer and then incubated with chicken anti-rabbit IgG, DyLight 594 conjugated secondary antibody (ImmunoReagents, Inc., Raleigh, NC) at a 1:500 dilution in binding buffer for 1 h at room temperature and washed 3× for 5 min in binding buffer prior to being imaged in DPBS. Images were obtained on a Nikon eclipse-TI2 inverted microscope equipped with a TI2-S-SS-E motorized stage, ORCA-Flash 4.0LT sCMOS camera, and NIS elements imaging software.

### Statistics

Experiments were performed at least 4 times using neutrophils from different donors on different days. Statistical analyses were performed by ANOVA or Student’s t-test using GraphPad Prism8 software. Differences of *p* < 0.05 were considered significant.

## Results

### Neutrophils From Invasive Candidiasis Patients Exhibit a Heightened Capacity for NET Formation

**Figure 1:**
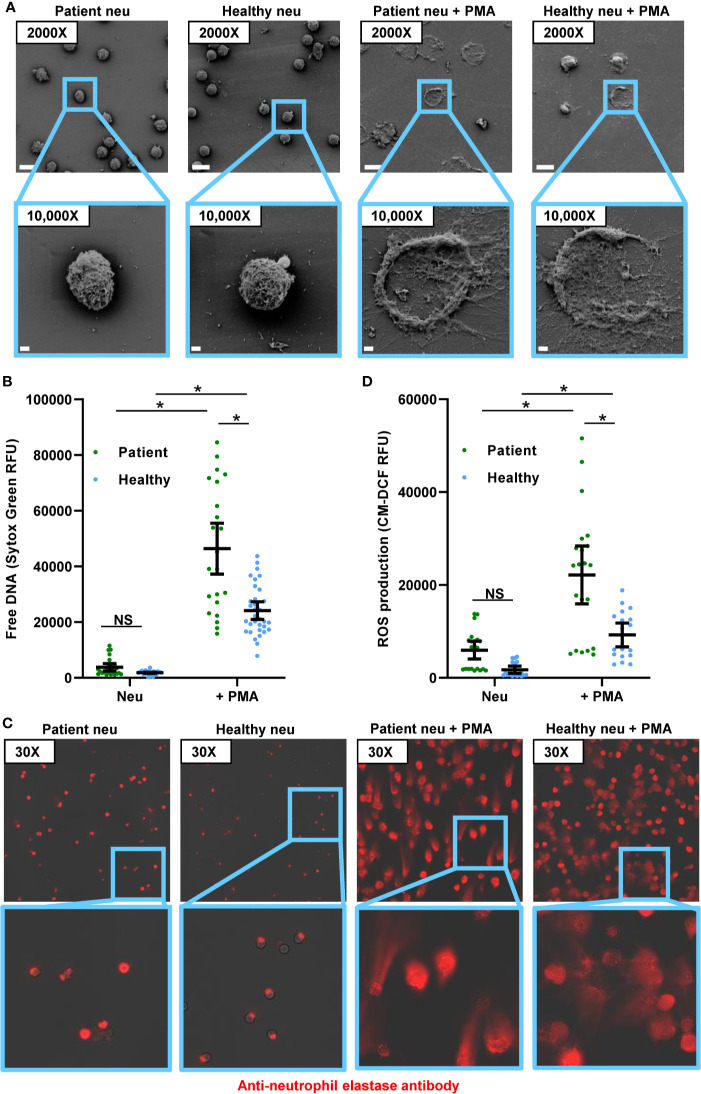
Comparison of PMA-induced NET formation and ROS production by neutrophils from healthy participants and patients with invasive candidiasis. **(A)** Scanning electron microscopy of patient and healthy neutrophils in the absence and presence of the NET stimulus PMA for 4 h. Measurement bars represent 10 µm and 1 µm for images taken at 2000X and 10,000X, respectively. **(B)** Patient and healthy neutrophils were incubated with or without PMA for 4 h and NET release was estimated by free DNA measurement following staining with cell-impermeable Sytox Green. **(C)** Neutrophils were incubated with or without PMA for 4 h, fixed, and stained with anti-neutrophil elastase rabbit primary antibody and chicken anti-rabbit IgG, DyLight 594 conjugated secondary antibody. **(D)** To measure ROS, neutrophils were pre-incubated with free radical sensor CM-H2DCFDA prior to incubation with or without PMA for 4 h, and fluorescence was measured. Statistical significance was analyzed by two-way ANOVA with Sidak’s multiple comparisons test, *p < 0.05, NS, not significant, mean with 95% confidence interval shown.

To delineate neutrophil responses for patients with invasive candidiasis, we collected blood from hospitalized patients and performed ex vivo neutrophil studies (**Table 1**). We also included neutrophils from healthy participants as a comparison, as prior *Candida*-neutrophil work has primarily utilized neutrophils from this population (6–8, 17–20, 23, 29, 30). In the absence of a stimulus, neutrophils from both study groups appeared rounded and NETs were only rarely observed by scanning electron microscopy over the course of 4 h (**Figure 1A**). To quantify NET release, we utilized a Sytox Green assay to measure free DNA (**Figure 1B**). Consistent with the scanning electron microscopy images, unstimulated neutrophils released very little free DNA. Neutrophils from patients with invasive candidiasis trended toward a higher background level of NET release, but this did not reach statistical significance. We next examined neutrophil responses to PMA, a strong inducer of NETs. Strikingly, in response to PMA, neutrophils from patients with invasive candidiasis released nearly 100% more DNA than neutrophils from healthy participants (**Figure 1B**). Scanning electron imaging revealed the formation of NET structures for both groups of neutrophils exposed to PMA, with more frequent NETs in the patient neutrophil group (**Figure 1A**).

During NET formation, neutrophil elastase translocates from the granules to the nucleus, where it cleaves histones and ultimately releases extracellularly with DNA as part of the web-like structures (30, 31). Using immunohistochemistry, we analyzed the location of neutrophil elastase. As described previously for unstimulated neutrophils from healthy people, neutrophil elastase remained intracellular and appeared punctate (**Figure 1C**). This is consistent with prior work that has shown an intracellular, granular localization for neutrophil elastase in resting neutrophils (**Figure 1C**) (30). While unstimulated neutrophils from patients also displayed intracellular neutrophil elastase, in a subset of neutrophils, staining was more diffuse. This suggested that neutrophil elastase may be translocating to the cytosol or nucleus, as has been described for NET release, even in absence of a stimulus. In response to PMA, both neutrophil groups exhibited web-like structures of extracellular neutrophil elastase, consistent with NET formation (**Figure 1C**). The extensive formation of NETs limited a semi-quantitative comparison.

ROS triggers the release of neutrophil elastase into the cytosol (30). Because ROS production is a key step in PMA-induced NET release, we considered the possibility that activation of this pathway might differ between neutrophils from patients and healthy participants (32). We prelabeled neutrophils with an oxidative stress indicator and measured ROS production over the course of 4 h in the presence and absence of PMA (**Figure 1D** and **Supplementary Figure 1A**). In the absence of a stimulus, neutrophils from patients with invasive candidiasis generated more than 3-fold higher ROS levels compared to neutrophils from healthy people. In response to PMA, ROS production mirrored the pattern observed for NET formation, with neutrophils from invasive candidiasis patients generating twice the levels of ROS (**Figures 1B, C**).

Because of the heighten ROS production observed for patient neutrophils, we considered if they may exhibit higher ROS levels at baseline. We found neutrophils from invasive candidiasis patients to generate approximately 2-fold higher levels of ROS compared to healthy participant neutrophils (**Supplementary Figure 1B**). Considering the role of ROS in PMA-induced NET formation, this trait may contribute to the increased NET formation observed for patient neutrophils. To further explore NET formation pathways, we examined histone citrullination. This enzymatic modification of histone arginine residues to citrulline residues mediates chromatin decondensation and NET formation (33). By immunofluorescence, we did not detect histone citrullination in healthy participant neutrophils (**Supplementary Figure 1C**). However, we did observe histone citrullination for a small number of neutrophils from patients with invasive candidiasis, suggesting some cells may have activation of NET formation pathways in the absence of a stimulus. Taken together, the studies reveal that neutrophils from patients with invasive candidiasis display an increased capacity for ROS production and PMA-induced NET formation that differs from neutrophils collected from healthy participants.

### Patient and Healthy Participant Neutrophils Respond Similarly to *C. albicans*

**Figure 2:**
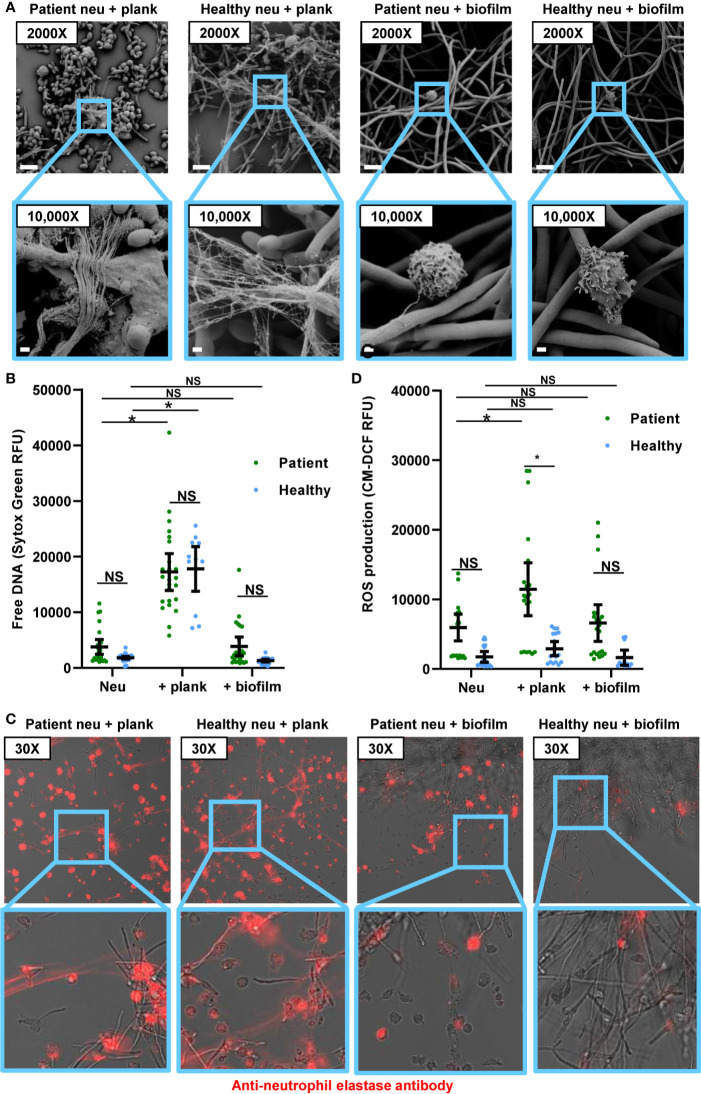
Response to planktonic and biofilm *C. albicans* for neutrophils from healthy participants and patients with invasive candidiasis. **(A)** Scanning electron microscopy of patient and healthy neutrophils co-incubated with planktonic or biofilm *C. albicans* for 4 h. Measurement bars represent 10 µm and 1 µm for images taken at 2,000× and 10,000×, respectively. **(B)** Patient and healthy neutrophils were co-incubated with planktonic or biofilm *C. albicans* for 4 h, and NETs were estimated by measurement of free DNA using Sytox Green. **(C)** Patient and healthy neutrophils were incubated with *C. albicans* planktonic cells or biofilm for 4 h, fixed, and stained with anti-neutrophil elastase rabbit primary antibody and chicken anti-rabbit IgG, DyLight 594 conjugated secondary antibody. Following fixation, neutrophils were stained for neutrophil elastase. **(D)** To measure ROS, neutrophils were pre-incubated with free radical sensor CM-H2DCFDA prior to incubation with planktonic or biofilm *C. albicans* for 4 h, and fluorescence was measured. Statistical significance was analyzed by two-way ANOVA with Sidak’s multiple comparisons test, *p < 0.05, NS, not significant, mean with 95% confidence interval shown.

We next questioned how neutrophils from invasive candidiasis patients respond to *C. albicans* and if these interactions differ from those that have been observed for healthy people. As previously reported, healthy participant neutrophils form NETs upon encounter with planktonic *C. albicans* (19, 20). Consistent with this, scanning electron microscopy imaging revealed healthy participant neutrophils forming web-like structures coating planktonic cells (**Figure 2A**), and the free DNA of NETs was detected at levels similar to PMA induction (**Figures 2B** and **1B**). Neutrophils from invasive candidiasis patients formed an equivalent amount of NETs upon encounter with planktonic *C. albicans* (**Figures 2A, B**). This was somewhat surprising in light of the heightened capacity of patient neutrophils for PMA-induced NET formation (**Figure 1B**). For both neutrophil groups, immunofluorescence imaging revealed strands of extracellular neutrophil elastase indicating the presence of NETs in response to planktonic *C. albicans* (**Figure 2C**). Upon examination of NET pathways following exposure to planktonic cells, we found that invasive candidiasis patient neutrophils generated 3-fold more ROS (**Figure 2D** and **Supplementary Figure 2A**). The finding that this elevation of ROS did not translate to increased NET formation by patient neutrophils is consistent with the involvement of ROS-independent pathways, as has been described for *C. albicans* (29, 31).

Upon co-culture with *C. albicans* biofilms, both groups of neutrophils failed to form NETs (**Figures 2A, B**). By scanning electron microscopy, neutrophils remained intact without the extrusion of extracellular structures (**Figure 2A**). Although neutrophils from invasive candidiasis patients released slightly more free DNA than neutrophils from healthy people, this was not above the background detected in the neutrophil only control (**Figure 2B**). Neutrophil elastase remained primarily intracellular for both neutrophil groups (**Figure 2C**). The staining appeared slightly more diffuse for the invasive candidiasis patient neutrophils, suggesting early translocation of neutrophil elastase from the granules, similar to the appearance of these neutrophils without a stimulus present (**Figure 1C**). Similarly, biofilms did not induce ROS above the background for either neutrophil control group (**Figure 2D**). Thus, the lack of NET production in response to *Candida* biofilm is a clinically relevant phenotype that translates to patients.

### *C. albicans* Biofilms Inhibit The Activity of Neutrophils From Patients With Invasive Candidiasis

**Figure 3:**
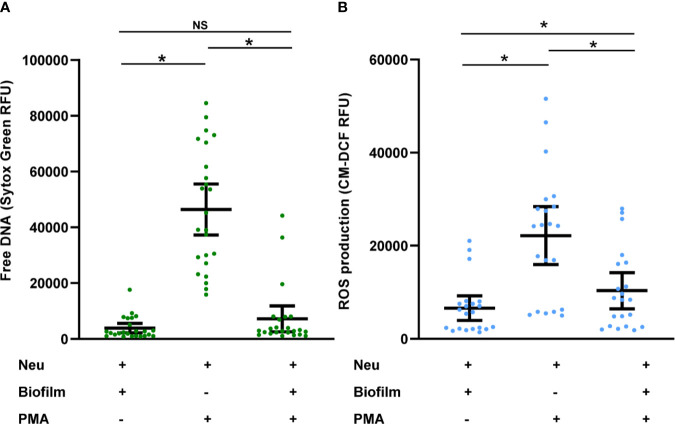
*C. albicans* biofilms inhibit PMA-induced activation of neutrophils collected from patients with invasive candidiasis. **(A)** Patient neutrophils were co-incubated with *C. albicans* biofilm in the absence or presence of PMA for 4 h and NET formation was estimated by measurement of free DNA using Sytox Green. **(B)** To measure ROS, neutrophils were pre-incubated with free radical sensor CM-H2DCFDA prior to incubation with *C. albicans* biofilm in the presence or absence of PMA for 4 h, and fluorescence was measured. Statistical significance was analyzed by one-way ANOVA with Tukey’s multiple comparisons test, *p < 0.05, NS, not significant, mean with 95% confidence interval shown.

Prior work with healthy participant neutrophils has shown that not only do *C. albicans* biofilms fail to induce NET formation, but also that NET release to other stimulants is suppressed by biofilm (18). Here, we wanted to determine if this phenotype translated to neutrophils from patients with invasive candidiasis. To test this, we measured NET formation by patient neutrophils in the presence of *C. albicans* biofilm and/or a stimulus for NET release (PMA). Consistent with our other experiments with patient neutrophils, PMA induced NET formation and biofilms did not (**Figure 3A**). When both biofilm and PMA were present, neutrophils failed to form NETs, indicating that biofilm suppresses PMA-induced NET formation. Similarly, biofilm formation inhibited PMA-induced ROS production (**Figure 3B** and **Supplementary Figure 2B**). These data support the finding that *C. albicans* biofilms suppress NET formation, even in the presence of a strong stimulant.

## Discussion

Invasive candidiasis is a widespread nosocomial infection with high mortality (1, 11). The vast majority of cases involve medical devices that become infected with *Candida* biofilms. While prior work has shown poor neutrophil responses to *Candida* biofilm, the role of these processes for patients with invasive candidiasis had not been elucidated (7, 8, 18, 23). Here, we characterize NET formation for patients with invasive candidiasis. With these patient neutrophils, we observe a heightened capacity for PMA-induced NET formation and activation of NET signalling pathways when compared to healthy participant neutrophils. Despite these different characteristics of patient neutrophils, we found that the neutrophils respond to *C. albicans* in a manner that mirrors healthy participant neutrophils. Both patient and healthy participant neutrophils formed NETs in response planktonic *C. albicans* but not upon encounter with *C. albicans* growing as a biofilm. These findings help explain why *Candida* biofilm infections are difficult to eradicate clinically. The studies also support the use of neutrophils from healthy people to model neutrophil responses during invasive candidiasis.

We were somewhat surprised to find that neutrophils from invasive candidiasis patients exhibited a heightened capacity to form NETs in response to PMA, but not to planktonic *C. albicans*. These differences suggest neutrophils from patients with invasive candidiasis might be primed for certain NET release pathways, but not others, depending on the stimuli (34). For example, PMA-induced NET release is ROS-dependent, whereas planktonic *C. albicans* can induce NETs through ROS-independent pathways (29, 31, 35, 36). We did observe a difference in ROS levels for patients and healthy participants, with patient neutrophils exhibiting elevated baseline ROS levels and higher ROS upon PMA treatment (**Figure 1D** and **Supplementary Figures 1A, B**). The current studies were not designed to determine the cause of the higher ROS production in patients. While this may be mainly due to candidiasis, our population included many patients with polymicrobial intra-abdominal infections (**Table 1**). Therefore, we are not able to exclude a contribution from bacterial infection. Furthermore, the studies did not include patients with serious bacterial infections or inflammatory conditions who did not have candidiasis. It is quite possible that neutrophils from these patient cohorts would also exhibit activation of NET pathways. Bacterial species, including Enterobacteriaceae and *Streptococcus* spp. have been shown to induce NETs through ROS-generating pathways (32, 37, 38). Furthermore, multiple factors may be influencing the neutrophil ROS production in these patients. However, despite the intrinsic heterogeneity of the patient population, the consistency of ROS elevation suggests this phenotype may be common among invasive candidiasis patients.

Prior work has shown that *C. albicans* biofilms inhibit NET release in healthy neutrophils, even in the presence of the potent stimulus (PMA) (18). Here, we show that biofilms also inhibit PMA-induced NET release in the neutrophils of patients with invasive candidiasis. This inhibition is striking in light of their increased baseline activation. Beyond this lack of activity against biofilms, suppression of neutrophils by *C. albicans* biofilm may impair the activation of other cells involved in innate and adaptive immunity (39, 40). Further study is needed to understand how fungal biofilms may influence immune responses to other pathogens in the clinical setting.

The subset of invasive candidiasis patients enrolled in our study are representative of larger cohort studies (1, 11, 41). For example, the study participants carried the diagnosis of immune-compromising diseases, received immunosuppressive drugs, and frequently required central lines (11, 41). However, as expected, we observed differences in co-morbidities, sites of infection, and *Candida* species. Despite the varied patient characteristics, we observed rather uniform neutrophil responses for all studies. This suggests that the findings may be broadly applicable to variety of patients with invasive candidiasis.

## Data availability Statement

The raw data supporting the conclusions of this article will be made available by the authors, without undue reservation.

## Ethics Statement

The studies involving human participants were reviewed and approved by the University of Wisconsin Internal Review Board. The patients/participants provided their written informed consent to participate in this study.

## Author Contributions

JK, CJ, and JN planned the study and designed the experiments. CJ, JK, and MB performed the experiments. JK, CJ, JC, and JN analyzed data, performed statistical analyses, and wrote the manuscript. JN supervised the work. All authors offered input to the manuscript and approved the final draft. All authors contributed to the article and approved the submitted version.

## Funding

This work was supported by the National Institutes of Health (R01 AI145939), the Burroughs Wellcome Fund (1012299), and the Doris Duke Charitable Foundation (2017074).

## Conflict of Interest

The authors declare that the research was conducted in the absence of any commercial or financial relationships that could be construed as a potential conflict of interest.
