# Supplementary material for: Neutrophils From Patients With Invasive Candidiasis Are Inhibited by Candida albicans Biofilms
Source: Front Immunol. 2020 Dec 3;11:587956. doi: 10.3389/fimmu.2020.587956 (PMC7747767; doi:10.3389/fimmu.2020.587956)
Supplement: Supplementary file 1 [file DataSheet_1.pdf]

Supplementary Material

Supplementary Figure 1. (A) Time courses for ROS production over 4 h. Patient and healthy participant neutrophils were pre-incubated with free radical sensor CM-H2DCFDA, and ROS production was measured every 30 min for 4 h in the presence and absence of PMA, mean with SEM shown. **(B)** Patient and healthy participant neutrophils ROS levels were measured without a stimulus. Statistical significance was analyzed by unpaired t-test, * $p<0.05$, mean with 95% confidence interval shown. **(C)** Neutrophils were stained with anti-histone 4-citrulline-3 antibody and anti-rabbit IgG, DyLight 594 conjugated secondary antibody.

Supplementary Figure 2. Time courses for ROS production over 4 h. Patient and healthy participant neutrophils were pre-incubated with free radical sensor CM-H2DCFDA, and ROS production was measured every 30 min for 4 h in the presence of *C. albicans* during planktonic or biofilm growth **(A)**, or in the presence of *C. albicans* biofilm and/or PMA **(B)**, mean with SEM shown.
