# Supplementary figures and images for: Neutrophils From Patients With Invasive Candidiasis Are Inhibited by Candida albicans Biofilms
Source: Front Immunol. 2020 Dec 3;11:587956. doi: 10.3389/fimmu.2020.587956 (PMC7747767; doi:10.3389/fimmu.2020.587956)

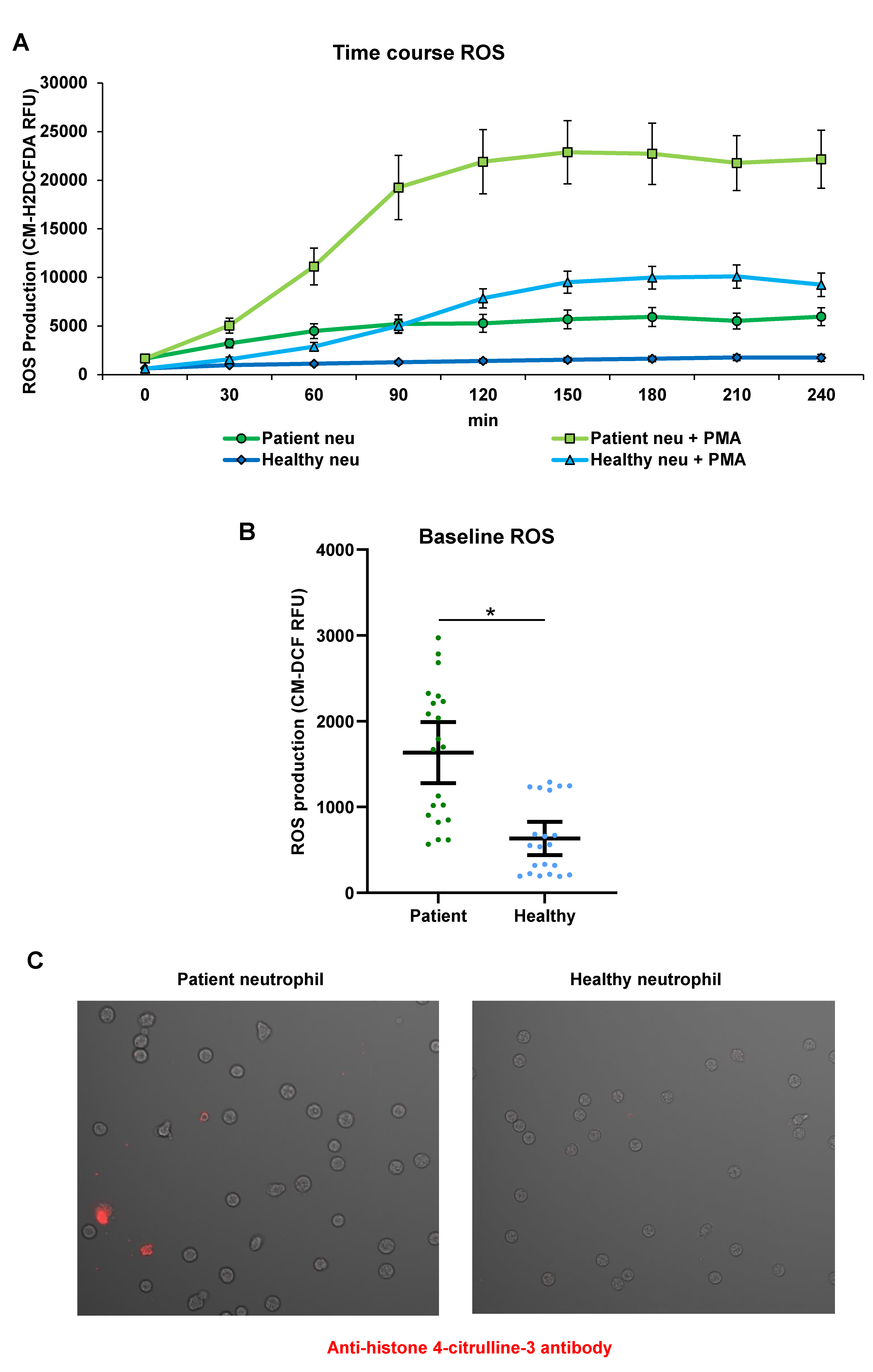

Supplement: Supplementary file 2 [file Image_1.tif]

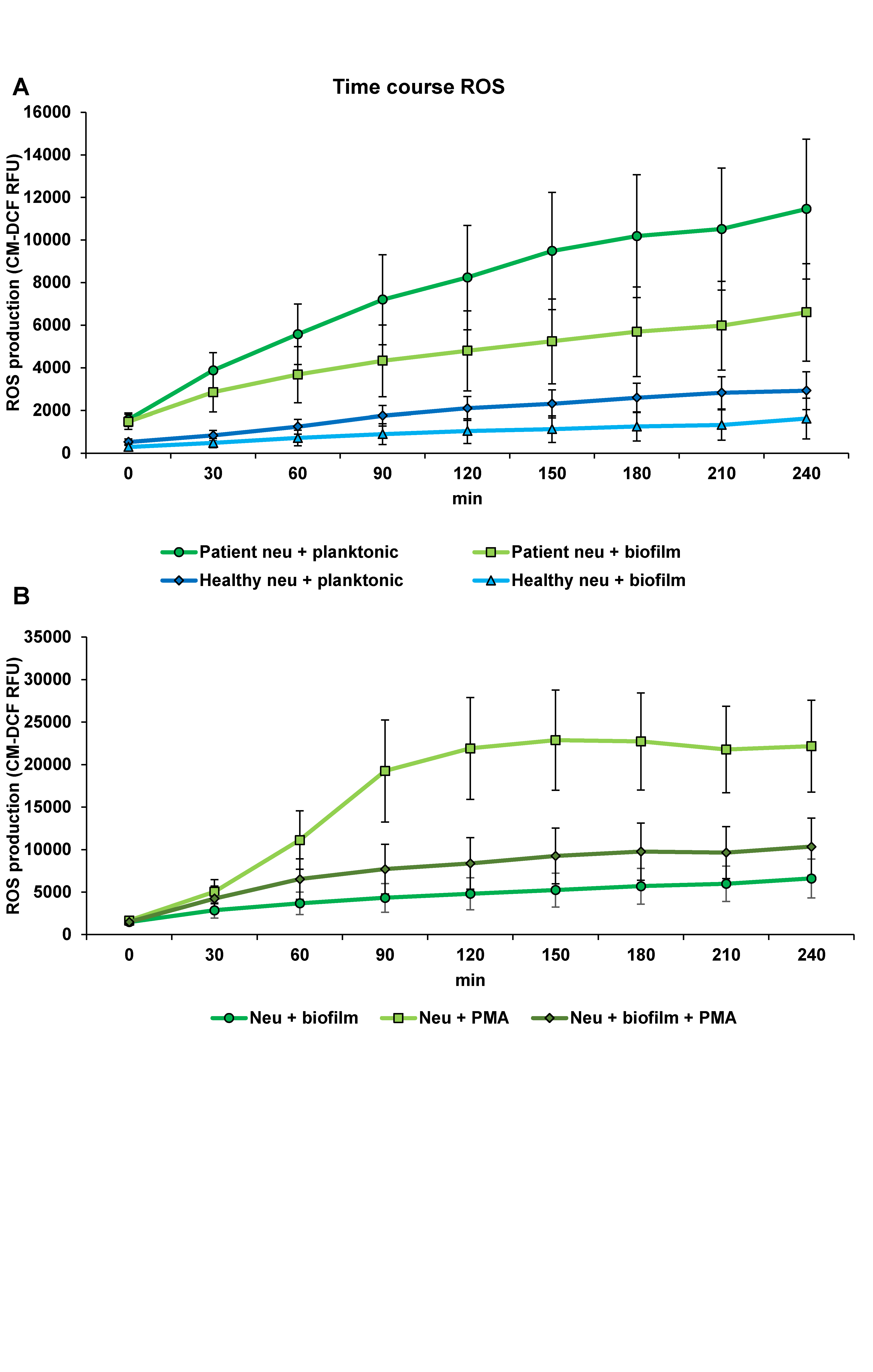

Supplement: Supplementary file 3 [file Image_2.tif]
